# Ethnomedicinal Investigation of Medicinal Plants of Chakrata Region (Uttarakhand) Used in the Traditional Medicine for Diabetes by Jaunsari Tribe

**DOI:** 10.1007/s13659-019-0202-5

**Published:** 2019-04-09

**Authors:** Ankit Kumar, Sonali Aswal, Ashutosh Chauhan, Ruchi Badoni Semwal, Abhimanyu Kumar, Deepak Kumar Semwal

**Affiliations:** 1 Research and Development Centre, Faculty of Biomedical Sciences, Uttarakhand Ayurved University, Harrawala, Dehradun, 248001 India; 2 Department of Biotechnology, Faculty of Biomedical Sciences, Uttarakhand Ayurved University, Harrawala, Dehradun, 248001 India; 3 Department of Chemistry, Pt. Lalit Mohan Sharma Government Postgraduate College, Rishikesh, Uttarakhand 249201 India; 4 Uttarakhand Ayurved University, Harrawala, Dehradun, 248001 India; 5 Department of Phytochemistry, Faculty of Biomedical Sciences, Uttarakhand Ayurved University, Harrawala, Dehradun, 248001 India

**Keywords:** Ayurveda, Diabetes, Herbal formulations, Traditional healers, Folk medicine

## Abstract

The Himalayan region is the treasure house of natural wealth, particularly of medicinal and aromatic plants. These plants are used by the Indian traditional healers for the past many centuries to treat various ailments such as skin disorders, asthma, diabetes, snake bite, fever, pain, eye diseases, diarrhoea, indigestion, jaundice, burn, wound, liver disorder, CNS disorders and urinary tract infection. The indigenous traditional knowledge of medicinal plants and therapies of various local communities has been lost due to changes in traditional culture and the introduction of modern technologies. Therefore, it is essential to explore the traditional knowledge of the indigenous medicinal plants mainly in such areas where there is a severe threat to natural vegetation owing to human inhabitation. The present study aimed to explore the medicinal plants of Chakrata region (Jaunsar–Bawar Hills), Uttarakhand, India used in the folk medicine for the management of diabetes by Jaunsari Tribe. In a comprehensive field survey, the information about the medicinal plants have been mainly collected from the traditional healers and other elderly people belong to the tribal community. All the information about the medicinal plants of the study area was documented in a field book. Various tools have been used to collect the samples for identification purpose and the authentication of the plants was done with the help of taxonomists. The literature on these plants was also searched from online (PubMed and Scopus) as well as from some textbooks and Ayurvedic classical texts. The present survey-based work described a total of 54 plants belonging to 47 genera and 30 families used in the traditional medicine for the management of diabetes in Chakrata region. The information gathered from the local community revealed that the plants are effective in diabetes and one can use most of them without consulting a practitioner or traditional healer. The literature revealed that most of the surveyed plants are already used in the preparation of various antidiabetic formulations such as Chandraprabha vati, Nishamalaki chunra, Amritamehari churna and Nisakathakadi kashayam along with various patent drugs which are frequently prescribed by the Ayurvedic practitioners in India. The present study explored the traditional as well as scientific knowledge on the antidiabetic plants used by the tribal community. The documented information on these plants can be further used by the scientific community to develop new drugs/formulations with the help of modern techniques.

## Introduction

Human has been directly or indirectly depending on the plants since time immemorial to fulfil his daily needs like food, oxygen, medicine and timber. Medicine is one of the essential necessities of a human, and the plants are the primary source of it. The plants have therapeutical importance to treat various kind of human and livestock ailments due to the presence of a variety of bioactive secondary metabolite. In different traditional medicinal systems like Indian and Chinese, these plants are used as a whole or their derived products in the form of different formulations. This ancient system of healthcare is also relevant and effective in the present time when technological progress has been drastically altered the individual’s lifestyle.

India is one of the mega-diversity countries recognised by the World Conservation Monitoring Centre in 2000. Its total geographical area is nearly 329 million hectares comprised of forests, grasslands, wetlands, coastal, marine and desert. India has an enormous ecological diversity ranging from sea level to the highest mountains. It represents 2.4% of world’s total geographical area with about 47,513 plants species out of 465,668 including microorganisms like virus, bacteria and fungi worldwide. The total numbers of species of angiosperms, bryophytes, pteridophytes and gymnosperms found worldwide are 268600, 16236, 12000 and 1021, respectively [1], whereas, in India, these are 18043, 2523, 1267 and 74, respectively. Among them, 4036, 629, 47 and 8, respectively are reported to endemic to India [2]. In India, about 280 medicinal plants belonging to 79 families are used by pharmaceutical industries to prepare different formulation used in Ayurvedic, Homeopathy, Unani, Siddha and even in Allopathic medicine, of which about 175 medicinal plants are found in the Himalayan region of India [3].

Diabetes mellitus is a metabolic disorder characterised by a high blood glucose level mainly due to the problem of inadequate insulin secretion by the pancreatic β-cells. A continuous high level of glucose in the blood can lead to serious complications such as neuropathy, retinopathy and nephropathy. In addition, one can become prone to heart attack and stroke if the glucose level is not maintained regularly. The patients of this deadly disease have been increased sharply in the past few years and still, the graph is rising rapidly. In 2013, the number of diabetic patients was about 422 million whereas this number was about 108 million in 1980. The cases of diabetes are recorded more in middle and low-income countries than in high-income countries. In 2015, about 1.6 million deaths were recorded due to diabetes mellitus. The majority of cases belong to type 2 diabetes mellitus, and these are about 80–90% of total diabetic cases. According to the World Health Organization [4], diabetes imputes the largest burden on the global economy and healthcare system. South-East Asia region which includes India, Bangladesh, Maldives, Nepal, Mauritius and Sri Lanka has one fifth (~ 84 million) of the total number of people with diabetes in the world, and hence called as the home of diabetes.

## Geography and Culture of the Study Area

Uttarakhand Himalaya is characterised as one of the micro-diversity centres of the Indian subcontinent. Chakrata, also known as Jaunsar–Bawar, is a hilly region in district Dehradun situated at a height ranging from 1500 to 2150 m. The Jaunsar region is the lower half while the snow-clad upper region is called Bawar. A tribal community, known as Jaunsari is inhabited in this region, and the native language spoken by them is Jaunsari, although Hindi is also spoken frequently. The culture of Jaunsari people is distinct from other natives of Uttarakhand and Himachal Pradesh, a neighbour hill state. The main occupation of this community is agriculture and animal husbandry. Geographically, it has rich vegetation and mostly covered by forest areas. The region is well-known for the medicinal plant diversity which includes many rare plants [5].

**Fig. 1:**
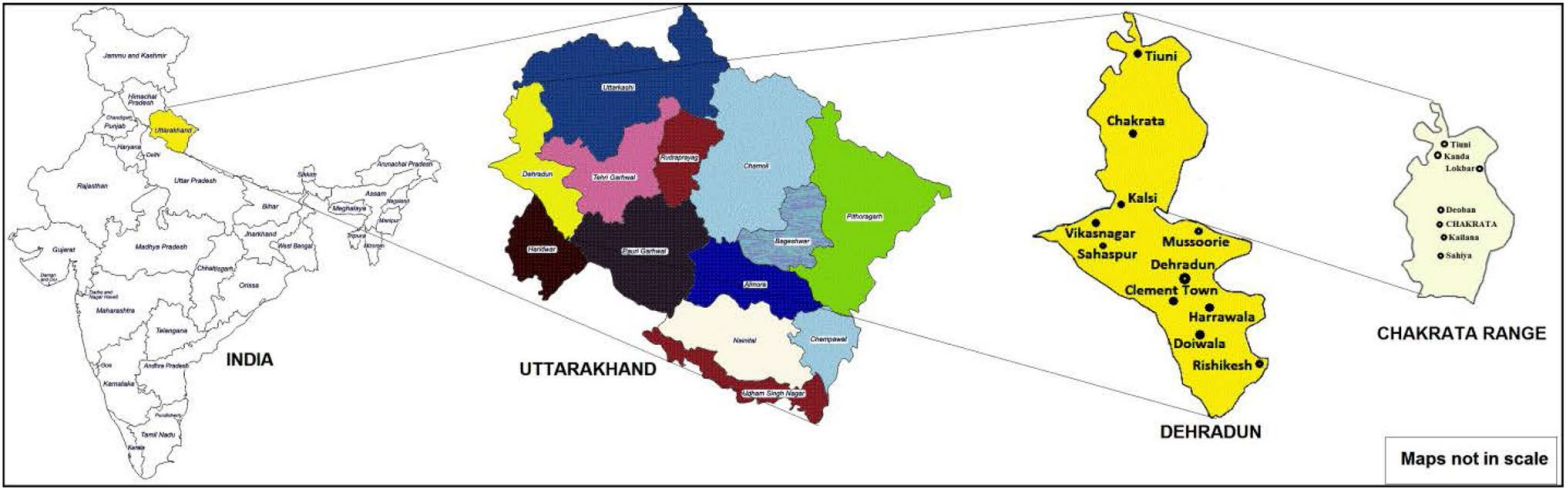
Geographical location of Chakrata region

The villages of Chakrata come under remote areas, therefore, less developed community centres and hospitals situated in the region. The insufficient medical facilities make compulsion to the people to use home remedies for all kind of health problems. Local traditional healers or Vaidyas works as the medical practitioner using native herbs based on traditional knowledge. Majority of the population believe in traditional medicine rather than modern treatment until unless a major problem occurs. The geographical location of the study area is shown in Fig. 1.

## Materials and Methods

### Data Collection

The literature based on the area, vegetation, population, social customs and culture of the study area has been studied before starting the field visits to the Chakrata region. A comprehensive field survey was conducted in the entire Chakrata region, including Tiuni, Deovan, Sahiya and Lokbar sites during February to March 2018. A total of four visits have been organised to get the maximum advantage of the tribal population to collect information about their plant resources for various purposes, mainly for the treatment of diabetes. The information was collected from the local tribal community, elder people, local traditional healers (Vaidyas) and some employees from the State Forest Department posted in this area. In addition to the general conversations with the informants, the questionnaires were also used to obtain information on the medicinal plants with their local names, parts used, mode of preparation and administration. The plants/parts used for the purpose of diabetes were also collected during the field visits for their taxonomical authentication. The collected plants/parts were kept in the deep fridge (− 20 °C) until their further use. After the identification, the plants/parts were pressed, dried and mounted on the herbarium sheets and preserved in the phytochemistry laboratory for future records.

### Data Analysis

During the survey, a total of 54 antidiabetic plants belonging to 47 genera and 30 families have been collected. The identification of the plants was done by Dr. M.R. Uniyal (Materia Medica Expert), Dr. A.K. Agrawal (Taxonomist, Govt PG College, Uttarkashi), Dr. Suresh Chaubey and Dr. R.C. Tiwari (Uttarakhand Ayurved University, Rishikul Campus, Haridwar). The identification also confirmed on basis of the local names of plants mentioned in the local Floras of Uttarakhand and the Himalayas. An in-depth literature search was conducted to confirm the scientific names of the antidiabetic plants. The updated botanical names of all plants were cross-checked from the Plant List, a working list of all plant species [6]. After identification, the scientific literature based on the antidiabetic potential of the medicinal plants was reviewed from the online database (PubMed and Scopus) and textbooks as well as from ancient Ayurvedic texts (Samhitas and Nighantus). Based on the literature survey, selected antidiabetic activity-based information has been included in the text for the scientific validation of traditional knowledge.

## Results and Discussion

Based on the field survey, about 120 species of medicinal plants were initially identified in the Chakrata region, of which, 54 plants (belonging to 47 genera and 30 families) were recorded as antidiabetic plants used in the traditional medicine. The highest number of anti-diabetic plants were documented in the family Lamiaceae (5) followed by Zingiberaceae (4), Amaryllidaceae (3), Apocynaceae (3), Compositae (3), Menispermaceae (3) and Moraceae (3). Out of 54 plants, 31 plants were found as cultivated and 15 as wild whereas 8 species were recorded as both cultivated and wild. The details of the above plants are given in Table 1, which contains information about the methods of administration and the selected pharmacological evidence of the antidiabetic plants with well-supported by relevant references. The results showed that most of the plants/parts are used in the form of decoction to treat diabetes.

**Table 1:** Traditionally used antidiabetic plants found in the surveyed area

Botanical names	Common names	Habit (habitat)/part used	Key bioactive constituents	Traditional method of use	Pharmacological evidence
Apiaceae					
*Anethum graveolens* L.	Shatapushpa (S), Soyu (H), Indian Dill (E)	Herb (C)/seeds	Essential oil (mainly contains d-carvone, limonene and α-phellandrene) [7]	An infusion of seeds in water is taken orally	(1) Aqueous seed extract (3.04 g/kg) decreased blood glucose level in ALX-induced diabetic mice when treated orally once a day for 15 days [8] (2) Hydro-alcoholic leaf extract (5% of total diet) reduced glucose levels, LDL-C, TC, AST, ALT, and fibrinogen in hyper-cholesterolemic rabbits in a 3 days oral treatment [9] (3) Hydro-alcoholic leaf extract (300 mg/kg) showed hypoglycemic effect similar to glibenclamide in ALX-induced type 1 diabetic rat [10] (4) Scientifically, it has antidiabetic effect in both humans and animals, and can be suggested for the diabetic patients [11]
*Carum carvi* L.	Kashmirajiraka (S), Krishna Jeera (A), Kala Jeera (H), Black cumin/Caraway (E)	Herb (C)/seeds	Essential oil (mainly contains carvone, limonene, anethole and carveol) [12]	An infusion of seeds in water is taken orally	Aqueous seeds extract (1 g/kg/day) decreased blood glucose level and alleviated the body weight loss of STZ-induced diabetic rat in a 21 days treatment [12]
Acanthaceae					
*Barleria prionitis* L.	Vjradanti (S) (H), Porcupine flower (E)	Shrub (C)/whole plant	Barlerinoside, barlerin, acetyl barlerin, barterin, scutellarin [13]	A decoction of the whole plant is taken with empty stomach	Alcoholic extract of the leaf (200 mg/kg) decreased blood glucose and glycosylated haemoglobin whereas increased insulin level and liver glycogen level in ALX-induced diabetic rat in a 14 days treatment [14]
*Justicia adhatoda* L.	Vasa/Vasaka (S), Adulsa/Adusa (H), Malabar nut (E), Bansoe (L)	Shrub (W)/leaves and roots	Vasicine (**13**), vasicinol (**14**), vasicinone [15]	(1) Juice of leaves is used with an empty stomach (2) A decoction of roots (~ 50 g) with cow milk (125 mL) is taken daily in the morning	Ethanol extract of leaves (100 mg/kg/oral) reduced in blood glucose level in ALX-induced diabetic rats in a 6 days treatment. The results were compared with glibenclamide (5 mg/kg). In addition, it showed a positive effect on the glucose tolerance, glycosylated haemoglobin, serum lipid profiles and body weight of diabetic rats [15]
Amaryllidaceae					
*Allium cepa* L.	Palandu (S), Piyaaz (H), Onion (E)	Herb (C)/bulbs	*S*-methylcysteine sulfoxide (**1**), allicin [16]	Two teaspoons of leaf juice are taken two times a day for several days	(1) Regular use of fresh bulbs (50 g/day) reduced insulin requirement in a diabetic patient from 40 to 20 units a day [17] (2) Fresh cut slices (100 g/day) reduced fasting blood glucose levels in type 1 and type 2 diabetic patients when compared with insulin and glibenclamide in 4 h. Besides, at the same dose, it reduced hyperglycemia induced by dextrose (75 g) in type 1 and type 2 diabetic patients [18] (3) Oral administration of *S*-methylcysteine sulfoxide (200 mg/kg) ameliorated diabetic conditions in ALX induced diabetic rats when compared to glibenclamide and insulin in a 2 months treatment [19]
*Allium sativum* L.	Lasuna (S/L), Lahasun (H), Garlic (E)	Herb (C)/bulbs	*S*-allylcysteine sulfoxide (alliin) (**2**), allicin, allyl sulfide [20]	Juice of its bulbs with the leaves of bael (*Aegle marmelos*) is taken in the morning	(1) Ethanol extract (500 mg/kg) decreased serum glucose, total cholesterol and triglycerides levels whereas increased serum insulin in STZ induced diabetic in rats in a 14 days oral treatment [21] (2) Alliin (200 mg/kg) ameliorated diabetic conditions in ALX induced diabetic rats when compared to glibenclamide and insulin in a 2 months treatment [19]
*Allium stracheyi* Baker	Jambu (S), Faran/Van faran (H), Jimbu pharan/Keer (L)	Herb (C)/whole plant	Essential oil (mainly contains 1,2 bis (methylthio) ethane, 2,4 dimethyl thiophene, dimethyl disulphide and dimethyl trisulphide) [22]	The crude bulbs or juice is orally taken for the treatment of diabetes	Not reported
Apocynaceae					
*Catharanthus roseus* (L.) G.Don	Sadapushpa (S); Sadabahar (H); Madagascar periwinkle/Vinca (E)	Herb (C)/whole plant	Vincristine, vinblastine [23]	A leaf infusion is taken twice a day for several days	(1) Ethanol leaf extract (200 and 400 mg/kg, p.o.) showed sugar lowering effect in STZ induced diabetic rats [24] (2) A suspension of dried leaf powder (100 mg/kg/day for 60 days) showed the antidiabetic effect on STZ induced diabetic rats [23]
*Gymnema sylvestre* (Retz.) R.Br. ex Sm.	Madhunashini/Meshashringi (S), Gurmar (H), Gymnema (E)	Climber (C)/whole plant	Gymnemic acid (**12**) [25]	(1) A tea or decoction prepared from fresh leaves or dried leaves powder (one teaspoon) is taken in the morning for a long time (2) A mixture of *Gymnema sylvestre* leaf (100 g) with the fruits of *Emblica officinalis*, *Belliric myrobolan*, *Chebulic myrobalan* (triphla), seeds of *Syzygium cumini*, *Momordica charantia* and *Trigonella foenum*-*graecum* (50 g each) is taken orally with water after breakfast	(1) The methanol extract of leaf and callus (200 mg/kg) increased the body weight, liver, pancreas and liver glycogen content in ALX-induced diabetic rats whereas gymnemic acid was found to regenerate β-cells in the diabetic rat (2) Ethanol leaf extract (200 and 400 mg/kg, p.o.) was found to possess a blood sugar lowering effect in STZ-induced diabetic rats [24]
Asparagaceae					
*Asparagus racemosus* Willd.	Shatapadi/Satamuli (S), Satavare (H), Butter milk root (E)	Climber (W/C)/tuberous roots	Shatvarin I–VI, aspargamine A [26]	(1) The tuberous roots are boiled with water and this decoction is taken orally once a day (2) Its dried root powder with *Gymnema sylvestre* leaves is taken twice a day, for 30 days	(1) The ethanol root extract (30 μg/ml) stimulated insulin secretion in isolated perfused rat pancreas, isolated rat islet cells and clonal β-cells in vitro [27] (2) Ethanol root extract (1.25 g/kg) improved glucose tolerance in diabetic rats with an oral sucrose load of 2.5 g/kg. It also suppressed postprandial hyperglycaemia after sucrose ingestion and reversibly increased unabsorbed sucrose content throughout the gut. Besides, it inhibited the absorption of glucose during in situ gut perfusion with glucose. The extract enhanced glucose transport and insulin action in 3T3-L1 adipocytes (3) Oral administration for 28 days decreased serum glucose, increased pancreatic insulin, plasma insulin, liver glycogen and total oxidant status in STZ induced diabetic rats [27]
Berberidaceae					
*Berberis aristata* DC.	Daruharidra (S), Daru haldi (H), Indian barberry (E), Kingod (L)	Shrub (W)/roots and stems	Berberine [28]	(1) A decoction of roots or stem bark with water (5–10 mL) is taken twice a day for 1–2 weeks (2) A decoction of *B. aristata* root and *Terminalia chebula* fruits is taken orally in the morning	Aqueous ethanol root extract (250 mg/kg) lowered the blood glucose level in ALX induced diabetic rats. It also increased the glucokinase and glucose-6-phosphate dehydrogenase activities and decreased glucose-6-phosphatase activity in diabetic rats which play a critical role in glucose homeostasis [29]
*Sinopodophyllum hexandrum* (Royle) T.S.Ying (Syn. *Podophyllum hexandrum* Royle)	Himalayan mayapple/American mandrake (E), Ban kakri (H/L)	Herb (C)/roots	Podophyllotoxin (podophyllin), quercetin 3-*O*-beta-d-galactopyranoside [30]	Half teaspoon of the powder of *A. parvi*flora leaves, *Aconitum heterophyllum* tuber and *Podophyllum hexandrum* roots is given twice a day in the morning and at night after meals up to 3 months	Not reported
Combretaceae					
*Terminalia bellirica* (Gaertn.) Roxb.	Bibhitaki (S), Baheda (H), Bellric Myrobalan (E), Bedu (L)	Tree (W/C)/fruits and bark	Ellericanin, gallic acid, ellargic acid [31]	(1) A tea/decoction prepared from fresh leaves or dried leaves powder (one teaspoon) is taken in the morning for a long time (2) A mixture of *Gymnema sylvestre* leaf (100 g) with the fruits of *Emblica officinalis*, *Belliric myrobolan*, *Chebulic myrobalan* (triphla), seeds of *Syzygium cumini*, *Momordica charantia* and *Trigonella foenum*-*graecum* (50 g each) is taken orally with water after breakfast (2) The extract/infusion of Triphala powder is taken with almonds in the morning	(1) The extract of dried fruits stimulated basal insulin output and potentiated glucose-stimulated insulin secretion in the clonal pancreatic beta-cell line (BRIN-BD11). It also displayed insulin-mimetic activity and enhanced insulin-stimulated glucose uptake in 3T3-L1 adipocytes. The extract also produced a decrease in starch digestion (in vitro) and inhibited protein glycation at a concentration of 50 mg/mL [32] (2) The oral administration of methanol extract of dried fruits (100 mg/kg) reduced blood glucose level in ALX induced diabetic rats in an 11 days study [33]
Compositae					
*Artemisia indica* Willd.	Pati (H)	Shrub (W)/aerial parts	Essential oil (mainly contains β-caryophyllene, germacrene D, caryophyllene oxide and cis-β-elemenone [34]	A decoction of leaves with water is used in the morning	The oral administration of hydro-methanolic extracts of aerial parts (200 and 400 mg/kg) and its chloroform fraction (200 mg/kg) for 15 days showed a reduction in blood glucose level of STZ induced diabetic rats [35]
*Artemisia roxburghiana* Wall. ex Besser	Roxburgh’s Wormwood (E), Kuranja/Kinid (L)	Herb (W)/aerial parts	Betulin (**4**), betulinic acid (**5**), artemisinin (**6**) [36]	The decoction of aerial parts is used either alone or in combination with the dried fruits of *Zizyphus jujube*	(1) The methanol extract of aerial parts (100 µg/mL), betulinic acid (IC_50_ 3.49 µM) and betulin (IC_50_ 4.17 µM) showed protein tyrosine phosphatase 1B (PTP1B) enzyme inhibitory activity in vitro [37] (2) The ethanol leaves extract (1 μg/mL) showed insulin secretagogue activity at a concentration of when tested for insulin release from insulinoma cell line (INS-1 cells) [38] (3) Artemisinin showed trypsin inhibitory activity [36]
*Artemisia vulgaris* L.	Dhamanaka (S), Douna (H), Indian Wormwood/Mugwort (E)	Herb (W)/whole plant	Essential oil (mainly contains α-pinen, menthol, β-eudesmol and spathulenol) [39]	A decoction of leaves with water is used in the morning	Oral administration of ethanol leaves extract (250 and 500 mg/kg) for 16 weeks reduced the renal impairment in diabetic nephropathy rats through anti-diabetic, anti-oxidant and protective morphological changes in STZ induced diabetes rats [40]
Euphorbiaceae					
*Mallotus philippensis* (Lam.) Müll.Arg.	Raktanga (S), Kampillaka (A), Kama (H), Monkey face tree (E), Ruena (L)	Tree (W)/fruits	Bergenin, friedelin, lupeol, betulin-3-acetate corotoxigenin rhamnoside, coroglaucigenin rhamnoside [41]	A decoction of fresh fruits is taken in the morning and evening before a meal	Oral administration of hydro-ethanol bark extract (200 and 400 mg/kg) for 30 days increased body weight and insulin level, and decreased blood glucose and glycosylated haemoglobin on STZ induced diabetic rats [42]
Lamiaceae					
*Mentha piperita* L.	Pudina (H), Peppermint (E)	Herb (C)/whole plant	Essential oil (mainly contains menthol, menthone, menthyl acetate and menthofuran [43]	Juice of leaves in water is taken twice a day for a long time	Oral administration of peppermint leaf juice (0.29 g/kg/day) for 21 days decreased the blood glucose level of ALX induced diabetic rats [44]
*Ocimum gratissimum* L.	Ajaka (S), Ram tulsi/Ban tulsi (H), Wild basil (E)	Herb (W/C)/whole plant	Essential oil (mainly contains eugenol and methyl eugenol) [45]	(1) A tea prepared from leaves is taken regularly for about 1 month (2) Fresh leaves are chewed after a meal	Aqueous leaf extract (250 mg/kg) showed anti-diabetic activity against fortified diet-fed STZ induced diabetic rats [46]
*Ocimum tenuiflorum* L.	Tulsi (S/H), Holy basil /Indian basil (E)	Sub-shrub (C)/whole plant	Essential oil (mainly contains β-ocimene, 1,8-cineole, camphor, Limonene, Linalool, Methyl-eugenol and β-Caryophyllene) [47]	(1) One teaspoon of powdered dried leaves with an equal ratio of *Azadirachta indica* leaves with water is taken thrice a day for 15 days (2) A herbal tea prepared with leaves is also used	(1) Ethanol leaf extract (200 and 400 mg/kg) showed a blood sugar lowering effect on STZ-induced diabetic rats [24] (2) Hydro-alcoholic leaf extract (250 and 500 mg/kg) showed the antidiabetic effect on STZ and nicotinamide induced diabetic rats [48]
*Ajuga parviflora* Benth.	Neelkanthi (A), Small-flowered Bugleweed (E), Bishkopra (L)	Herb (W/C)/whole plant	Ajugarin I, deoxyajugarin I, ajugarin I, chlorohydrin, 3β-acetoxy-clerodinin C [49]	Half teaspoon of the powder of *A. parviflora* leaves, *Aconitum heterophyllum* tuber and *Podophyllum hexandrum* roots is given twice a day in the morning and at night after meals up to 3 months	Ethanol extract of the whole plant showed in vitro inhibitory effect on alpha-amylase with an IC_50_ value of 110.18 µg/mL. In addition, the extract (60 mg/kg) showed anti-diabetic by reducing blood sugar level and body weight in ALX-induced diabetic rats [50]
*Vitex negundo* L.	Nirgundi (S/H), Five-leaved chaste (E), Shimaloo/Somi (L)	Shrub (W)/whole plant	Casticin, isoorientin, chrysophenol D, luteolin, essential oil (mainly contains sabinene, linalool, terpinen-4-ol, β-caryophyllene, α-guaiene and globulol) [51]	The decoction of the whole plant is taken twice a day after meal	(1) A crude polysaccharide fraction (50 mg/kg) isolated from the leaves reduced food intake, body weight and fasting glucose levels in db/db mice in a 7 days study [52] (2) An iridoid glucoside (50 mg/kg) isolated from *V. negundo* leaves reduced the levels of plasma glucose, glycosylated haemoglobin and increased in the levels of insulin and haemoglobin in STZ induced diabetic rats in a 30 days study when compared with the glibenclamide (5 mg/kg) which was used as a positive control. Besides, it increased glycolytic enzymes level and glycogen content and decreased the levels of gluconeogenic enzymes in the liver of diabetic rats [53]
Leguminosae					
*Bauhinia variegata* L.	Kachnara (S), Kachnar (H), Camel’s foot tree (E)	Tree (W/C)/stem bark and flowers	Naringenin, quercetin 3-methyl ether, luteolin, rutin, isoquercitrin, daucosterol, 2′-hydroxy4′,6′-dimethoxy-3,4-methylenedioxy chalcone, kaempferol-3-*O*-glucoside [54]	About 10 mL of the bark juice (overnight soaked with water) is taken in the morning before a meal	Aqueous leaves extract (500 and 1000 mg/kg) decreased plasma glucose, cholesterol, triglyceride, creatinine and blood urea nitrogen level of both type 1 and type 2 diabetes in high-fat diet and STZ induced diabetic rats in a 28 days study. Besides, it decreased the necrotic changes in the pancreatic tissue [54]
*Trigonella foenum*-*graecum* L.	Methika (S), Methi (H/L), Fenugreek (E)	Herb (C)/whole plant	Sotolone (**16**), diosgenin (**20**), trigonelline (**21**), 4-hydroxyisoleucine [55]	(1) The overnight soaked seeds (2 teaspoons) are used in the morning (2) Seeds and leaves are included in food preparations to treat diabetes (3) Seeds powder is used with cold water (4) The mixture of grind fruits of Triphla and seeds of *Syzygium cumini*, *Momordica charantia* and *Trigonella foenum*-*graecum* (50 g each) mixed with *Gymnema sylvestre* (100 g) is taken orally with water in the morning after breakfast	(1) In vitro study demonstrated that the aqueous seed extract (100 ng/mL) increased the glucose uptake through upregulation of mRNA expression levels of glucose transporter (GLUT-2) and sterol regulatory element binding protein (SREBP1C) in HepG2 cells. It also increased the activities of glycogen kinase and glycogen synthase enzymes by imparting modifications to downstream insulin signalling pathways. In addition, its seeds extract (5–20 µg/mL) imparted insulin mimicking properties by increasing the intracellular creatinine levels in L6C11 muscle cells (2) Diosgenin (1–10 µM) isolated from seeds improved the hyperglycemia and diabetic condition by promoting the adipocyte differentiation of 3T3-L1 cells in vitro. It increased the mRNA expression levels of CCAAT/enhancer-binding protein (C/EBP), peroxisome proliferator activated receptor-γ (PPAR-γ), and its target genes adipocyte protein 2, lipoprotein lipase, and glucose transporter-4. Besides, 4-hydroxyisoleucine and sotolone isolated from seeds were also found anti-diabetic at various concentrations [55]
Malvaceae					
*Bombax ceiba* L.	Shalmali (S), Semal (H), Silk Cotton tree (E)	Tree (W/C)/fruits and flowers	Shamimin, isohemigossylic acid lactone-2-methyl ether, ceibanaphthaquinone, hentriacontane [56]	(1) One teaspoon of powdered flowers and fruits is used twice a day after meal (2) A dish prepared from its fruits is useful in diabetes	The ethanol leaves extract (140 and 280 mg/kg) showed hypoglycemic activity on STZ-induced diabetic rats and alleviated dyslipidemia. The extract had a considerable protective effect on pancreatic β-cells and a stimulatory effect on insulin secretion from the remaining pancreatic β-cells [57]
Meliaceae					
*Azadirachta indica* A.Juss.	Nimba (S), Neem (H), Indian Lilac (E)	Tree (C)/all parts	Avicularin (**7**), castalagin (**9**), nimbin, nimbidin, azadirachtin, nimbinin [58]	(1) Juice of stem bark is mixed with an equal amount of fresh cow milk, taken for 7 days early in the morning with empty stomach (2) Dried and powdered leaves alone or in combination with black pepper are given thrice a day for 15 days (3) Roasted bark powder with buttermilk once in a day for 40 days (4) Fresh leaves (4–5) or leaves paste (one teaspoon) are useful in diabetes	(1) Acetone, ethanol and water extract of leaves showed in vitro α-amylase inhibition at different concentrations range from 1.25 to 10 mg/mL, aqueous extract was found least active with an IC_50_ value of 9.15 mg/mL. However, these extracts were found poorly active against α-glucosidase inhibition [59] (2) Oral administration of ethanol leaves extract (200 and 400 mg/kg) showed blood sugar lowering activity in diabetic rats [24] (3) Chloroform leaves extract showed oral glucose tolerance activity and reduced the intestinal glucosidase activity in STZ-induced diabetic mice. It increased glucose-6-phosphate dehydrogenase activity and hepatic, skeletal muscle glycogen content, after 21 days of treatment. The study revealed the regeneration of insulin-producing cells and a corresponding increase in the plasma insulin and c-peptide levels with the treatment [60]
*Melia azedarach* L.	Mahaneem (S), Bakain (H), Persian lilac (E)	Tree (W/C)/all parts	Azedarachic acid (**15**), nicotinic acid, gallic acid, para-coumaric acid, vanillic acid, chlorogenic acid, syringic acid, caffeic acid, ferulic acid, fatty acids (caproic, palmitic, stearic, oleic, linoleic, and linolenic acid) [61]	The decoction of aerial parts is taken in the morning	(1) Bioassay-guided fractions and isolates of fruits and leaves showed inhibitory effects on protein tyrosine phosphatase-1B enzyme as well as glucose uptake stimulation on C_2_Cl_2_ myoblasts cells in vitro [62] (2) Aqueous leaf extracts (300, and 400 mg/kg, intraperitoneal) displayed anti-diabetic on type 2 mice [63]
Menispermaceae					
*Cissampelos pareira* L.	Patha (S), midwife’s herb (E), Padi/Parh (L)	Climber (W/C)/whole plant	Pelosine, l-curine, hayatinine, hayatidine, cissampareine, cissamine, dicentrine, cycleanine, insularine, cycleanine, nuciferine, bulbocarpine, corytuberine, magniflorine, norimeluteine, pareitropone, berberine (8), reserpine [64]	The dried root powder (half teaspoon) is taken with water once a day for 40 days	The hydro-alcoholic leaves extract (200 and 400 mg/kg, p.o.) showed anti-diabetic activity by decreasing fasting blood glucose and increasing the body weight of on STZ-induced diabetic rats when compared to glibenclamide (5 mg/kg) [65]
*Stephania glabra* (Roxb.) Miers	Purha (H), Gindaru/Kuti (L)	Climber (W/C)/whole plant	Gindarine, gindaricine, gindarinine, cycleanine, columbamine, jatrorrhizine, magnoflorine, stepharanine, dehydrocorydalmine, pronuciferine, corydalmine, stepholidine, roemerine, palmatrubine, *N*-desmethylcycleanine, capaurine, corynoxidine, 4ʹ,5,7-trihydroxy-8-C-glucosylisoflavone, glabradine, gindarudine, 11-hydroxypalmatine (19), 8-(4ʹ-methoxybenzyl)-xylopinine, cepharamine, tuduranine [66]	The dried tuber powder (half teaspoon) is taken with water once a day for 40 days	Oral administration of 11-hydroxypalmatine (50 and 100 mg/kg), isolated from tubers, reduced blood glucose level in ALX-induced diabetic mice [67]
*Tinospora sinensis* (Lour.) Merr.	Guduchi (S), Giloy (H), Heart-leaved moonseed (E)	Climber (W/C)/whole plant	Giloin, giloinin giloinsterol, tinosporine, magnoflorine, tembetarine, berberine (**8**), choline, palmatine, jatrorrhizine, beberine, tembeterine, choline [68]	(1) One cup of aqueous infusion of stem buds is taken twice a day before a meal (2) 1 mL juice of *T. sinensis* with 5 g pulps of *Aloe vera* is taken for several days (3) 25 mL of stem juice in an equal amount of water is taken twice a day before the meal	The oral administration of aqueous root extract (5 and 7.5 g/kg) caused a reduction in the glucose level of blood and urine of ALX induced diabetic rats. It also decreased hepatic glucose-6-phosphatase and serum acid phosphatase, alkaline phosphatase, and lactate dehydrogenase in diabetic rats [69]
Moraceae					
*Ficus auriculata* Lour.	Fagoora/Timla (H), Roxburgh fig (E)	Tree (W/C)/fruits and leaves	Betulinic acid, lupeol, stigmasterol, bergapten, scopoletin [70]	Infusion (half cup) of leaves is taken in the morning	The methanol leaves extract (300 and 600 mg/kg) produced a significant reduction in blood glucose level in STZ induced diabetic mice. It also ameliorated the histological damage of Islets of Langerhans in the pancreas caused by STZ [71]
*Ficus religiosa* L.	Ashwattha (S), Peepal (H), Sacred fig (E)	Tree (W)/bark, leaves, stem, fruits and latex	Bergapten, bergaptol, lupeol, β-sitosterol, stigmasterol, lanosterol, campesterol, octacosanol, methyl oleonate, lupen-3-one [72]	The decoction (25 mL) of bark is used in the morning after meal	The aqueous bark extract showed an anti-diabetic effect in STZ-induced diabetic rats by decreasing the blood glucose, serum triglyceride and total cholesterol levels, and increasing serum insulin, body weight and glycogen content in the liver and skeletal muscle. The extract up to 2000 mg/kg was considered safe [72]
*Morus alba* L.	Tula/Brahmandaru (S), Sahatoot (H), White mulberry (E), Tut/Tutri (L)	Tree (C)/leaf, root, bark and fruits	1-Deoxy-nojirimycin, isoquercitrin, astragalin (17), rutin [73]	One cup of tea prepared from leaves (or root/bark) is used with an empty stomach	(1) Ethanol leaves extract (600 mg/kg) has therapeutic effects in STZ induced diabetic rats and can restore the diminished β cell numbers in a 35 days study [74] (2) Fruit polysaccharides fractions showed marked antihyperglycemic and antihyperlipidemic activities and repaired the damaged pancreatic tissues of the diabetic rats [75]
Phyllanthaceae					
*Phyllanthus emblica* L.	Amalaki (S), Amla (H), Indian gooseberry/Emblic myrobalan (E), Aunla (L)	Tree (C)/fruits	Gallic acid, glucogallin, 3,6-di-*O*-galloyl-d-glucose, 1,6-di-*O*-galloyl-βd-glucose, chebulinic acid, chebulagic acid, corilagin, 3-ethylgallic acid, isostrictiniin, l-malic acid 2-*O*-gallate, mucic acid 2-*O*-gallate, mucic acid 1,4-lactone 2-*O*-gallate, phyllaemblicin A–C, phyllaemblic acid, phyllaemblic acid B and C, phyllaemblicin D, 2-carboxylmethylphenol 1-*O*-βd-glucopyranoside, 2,6-dimethoxy-4-(2-hydroxyethyl)phenol 1-*O*-βd-glucopyranoside;ascorbic acid, emblicanin A and B, punigluconin, pedunculagin, [76]	(1) The mixture of grind fruits of Triphla and seeds of *Syzygium cumini*, *Momordica charantia* and *Trigonella foenum*-*graecum* (50 g each) mixed with *Gymnema sylvestre* (100 g) is taken orally with water in the morning after breakfast (2) The infusion of Triphala powder (fruits of *P. officinalis*, *T. chebula* and *T. bellirica*) is taken twice a day before a meal	The oral administration of methanol fruits extract (100 mg/kg) reduced blood glucose level in ALX induced diabetic rats within 4 h at single doses and at multi-doses up to 11 days [33]
Pinaceae					
*Cedrus deodara* (Roxb. ex D.Don) G.Don	Indradaru (S), Devdara (H), Himalayan cedar (E), Diwar/Kelon (L)	Tree (W/C)/heartwood, bark, leaves	Himachald, allohimachalol, himadarol, centdard, isocentdarol, dewarene, dewardiol, dewarenol, taxifolin, cedeodarin, dihydromyricetin, cetrin, cedrinoside [77]	A half cup of the decoction of the bark is taken twice after the meal	The ethanol bark extract (250 and 500 mg/kg) decreased blood glucose level, SGPT, SGOT, cholesterol and triglycerides in STZ induced diabetes mice. At the dose of 500 mg/kg, its effect was found comparable to that of glibenclamide (10 mg/kg). The extract also enhanced the regeneration of Islet of Langerhans in the pancreas and restoration of the normal cellular size of diabetes mice [77]
Plantaginaceae					
*Digitalis purpurea* L.	Hritpatri (S), Tilpushpi (H), Foxglove (E)	Herb (C)/leaf	Digoxigenin, digitonin (11), digitoxin, digoxin, ouabain, oleandrin, proscillaridin [78]	Leaf powder is taken in the morning but in less quantity under the observation of a traditional health practitioner. Higher concentration is believed to be toxic. Not recommended for children below 12 years	Digitonin (15 mg/kg) isolated from seeds improved glucose tolerance in high-sucrose-induced hyperglycaemic rats [78]
*Plantago ovate* Forssk.	Ashwagola (S), Isabgol (H), Spogel seeds/desert Indianwheat/Psyllium (E), Sabgul (L)	Shrub (C)/seeds and seed husk	Xylose, lilonic acid, galacturonic acid arabinose, uronic acid [79]	Seed and husk are taken twice a day after meal	The oral administration of aqueous husk extract (500 mg/kg) improved glucose tolerance in type 1 and type 2 diabetic rats. It suppressed the postprandial blood glucose level and retarded small intestinal absorption without inducing the influx of sucrose into the large intestine when administered with a sucrose solution [80]
Poaceae					
*Hordeum vulgare* L.	Yava (S), Jav/Jau (H), Barley (E)	Herb (C)/seeds	Caffeic acid, *p*-coumaric acid, 8,5’-diferulic acid, catechin-7-*O*-glucoside, saponarin, catechin, procyanidin B3, procyanidin C2, prodelphinidin B3, hordenine [81]	One to two pieces of bread prepared from the flour of Barley and Chana (*Cicer arietinum* L.) is taken per day for several days	The oral administration of aqueous seeds extract reduced the fasting serum glucose level of STZ-induced diabetic rats in a 28-days study [82]
Polygonaceae					
*Rheum australe* D. Don (Syn. *Rheum emodii* Wall. ex Meisn.)	Amlaparni (S), Dolu/Revandchini (H), Himalayan rhubarb (E)	Herb (C)/underground parts	Chrysophanol, physcion, rhein, emodin, aloe emodin [83]	Decoction (25 mL) of underground stem and root is taken in the morning after a meal for several days	Chrysophanol, physcion, rhein, emodin, and aloe emodin (2 mg/kg each) isolated from rhizomes showed antidiabetic activity in STZ induced diabetic rats; aloe emodin exhibited maximum blood glucose lowering effect. In the α-glucosidase inhibitory assay, only emodin was found active with the inhibitory effect of 93% [83]
*Terminalia chebula* Retz.	Haritaki (S), Harad (H), Chebulic myrobalan (E)	Tree (C)/fruits and bark	Chebulagic acid, chebulinic acid, Terflavin B [31]	(1) The mixture of grind fruits of Triphla and seeds of *Syzygium cumini*, *Momordica charantia* and *Trigonella foenum*-*graecum* (50 g each) mixed with *Gymnema sylvestre* (100 g) is taken orally with water in the morning after breakfast (2) The infusion of Triphala powder is taken in the morning after meal	The oral administration of methanol fruits extract (100 mg/kg) significantly reduced blood glucose level ALX induced diabetic rats within 4 h at single doses and at multi-doses up to 11 days [33]
Ranunculaceae					
*Aconitum heterophyllum* Wall. ex Royle	Ativisha (A/S), Atish (H), Indian Atish (E)	Herb (C)/roots	Aatisine, dihydroatisine, hetisined, heteratisine, 12-secohetisan-2-ol, *N*-succinoylanthranilate, atesinol 6-benzoylheterastine, *N*-diethyl-*N*-formyllaconitine, methyl aconitine, aconitine, anthorine [84]	The powder of Atish roots, *Ajuga parviflora* leaves and *Podophyllum hexandrum* roots is given twice a day (half teaspoonful) early in the morning and at night after meals up to 3 months	The oral administered of methanol root extract (200 mg/kg/day for 28 days) increased the levels of plasma glycoproteins and also decreased the level of sialic acid and elevated levels of hexose, hexosamine and fructose in the liver and kidney of STZ induced diabetic rats [85]
Rosaceae					
*Rubus ellipticus* Sm.	Golden/yellow Himalayan raspberry (E), Hinsar/Hisalu (L)	Shrub (W)/fruits and roots	Gallic acid, catechin, chlorogenic acid (**18**), caffeic acid [86]	One teaspoon powder of roots is taken twice a day for 1 month	The petroleum ether, ethanol and aqueous extracts of fruits (200 mg/kg each) exhibited antidiabetic activity using GTT in ALX induced diabetes rats [87]
Rubiaceae					
*Rubia cordifolia* L.	Aruna (S), Manjistha (A), Manjeeth/Majith (H), Indian madder (E), Charchora (L)	Climber (C)/roots	Cordifoliol, cordifodiol, rubiacordone, purpurin, alizarin [88]	The infusion (25 mL) of roots is taken once a day for 40 days	The oral administration of aqueous root extract (1 g/kg/day for 8 weeks) showed an anti-hyperglycemic effect in STZ induced diabetic rats [89]
Rutaceae					
*Zanthoxylum armatum* DC.	Tejohva/Tejovati (S), Tejbal (H), Yellow wood Tree timaru (L)	Shrub (W)/bark fruit and root	Essential oil (mainly contains linalool and limonene), armatamide [90]	One teaspoon powder of roots or stems is taken twice a day for 1 month	Methanol extracts of the fruits, leaves and bark showed α-glucosidase inhibitory activity in vitro. All the extracts at 500 mg/kg for 15 days were found to decrease fasting blood glucose levels in ALX induced diabetic mice [91]
Saxifragaceae					
*Bergenia ciliata* (Haw.) Sternb.	Pashanabheda (S), Patharchat (H), Hairy Bergenia (E)	Herb (W)/whole plant	Bergenin Bergenin, catechin, gallic acid, tannic acid [92]	Decoction (half cup) of aerial parts is taken before breakfast	(1) The hypoglycemic activity of aqueous, ethanol, butanol, chloroform, ethyl acetate and hexane extracts of leaves and root were evaluated by measuring blood glucose level, at the dose of 200 mg/kg (2) The aqueous and ethanol extracts of leaves and ethanol and hexane extracts of root at 200 mg/kg caused up to 70% decrease in blood glucose level in STZ-induced diabetic rats [93]
Solanaceae					
*Nicotiana tabacum* L.	Gucchaphala (S), Tamakhu (H), Tobacco (E)	Sub-shrub (C)/dried leaves	Nicotine, nornicotine, anatabine, rutin, chlorogenic acid, anabasine, myosmine, cotinine, tabacinine, tabacine, 2,3,6-trimethyl-1,4-naphthoquinone, 2-methylquinone, 2-napthylamine, propionic acid, anthalin, anethole, acrolein, cembrene, choline, nicotelline, nicotianine, pyrene [94]	(1) The aqueous decoction (25 mL) of leaves is taken in the morning after meal (2) The aqueous decoction of leaves with *Bidens pilosa* seeds, *Alstonia congensis* roots and potash is taken every 3 days interval before breakfast	The aqueous leaves extract was found an effective inhibitor of α-amylase (IC_50_ 5.70 mg/mL) while acetone leaves extract displayed remarkable inhibitory effect on α-glucosidase (IC_50_ 4.50 mg/mL) in vitro [95]
*Solanum virginianum* L.	Kantakari (S), Pili kateri (H), Yellow-fruit nightshade (E), Konkaru (L)	Herb (W)/whole plant	Lupeol, solasodine and its glycoside, tomatidenol, diosgenin (**20**), carpesterol, α-solamargine [96]	The aqueous decoction (25 mL) of aerial parts is taken in the morning after meal	(1) The methanol leaves extract (200 mg/kg) showed antidiabetic activity against ALX induced diabetic rats [97] (2) Lupeol, isolated from stem bark suppressed the progression of diabetes in rats. Its treatment caused decreasing glycated haemoglobin, serum glucose and nitric oxide in a 21 days study [98] (3) The aqueous fruits extract showed hypoglycemic activity in diabetic rats [99]
Taxaceae					
*Taxus baccata* L.	Manduparni (S), Talispatra (A), Gallu/Thuno (H), Himalayan yew (E)	Tree (W/C)/bark and seed	Taxol, taxine A and B, baccatin III and V, ephedrine [100]	The aqueous decoction (25 mL) of bark and seeds is taken twice a day for 40 days	Aqueous methanol leaves extract inhibited rat intestinal sucrase, maltase and porcine pancreatic α-amylase by 17.3, 35.4 and 25.8%, respectively at the concentration of 15 mg/mL in vitro [101]
Urticaceae					
*Urtica dioica* L.	Vrscikali (S), Stinging nettle/Nettle leaf (E), Bichchhu ghaas (H), Kandadli (L)	Herb (W)/whole plant	β-Sitosterol, ferulic acid, dotriacotane, erucic acid, ursolic acid, scopoletin, rutin, quercetin, *p*-hydroxylbenzalcohol [102]	The juice or tea prepared from leaves is taken once a day after meal	Aqueous leaves extract (300 mg/kg) showed a reduction in the blood glucose level in STZ-induced diabetic rats using a GTT assay [103]
Xanthorrhoeaceae					
*Aloe vera* (L.) Burm.f.	Gritkumari (S), Gheekuwar (H), Indian aloe (E)	Herb (C)/leaves	Aloin, aloesin, emodin, aloesone lophenol (**3**), 24-methyl-lophenol, 24-ethyl-lophenol, cycloartanol, 24-methylene-cycloartanol [104]	(1) The leaf gel (100 g) with water and lemon juice is taken in after breakfast for several days (2) One mL juice of *Tinospora sinensis* with 5 g pulps of *Aloe vera* is taken for several days	(1) The oral administration of leaves extract (300 mg/kg/day for 3 weeks) to STZ-induced diabetic rats showed restoration of blood glucose levels with a concomitant increase in insulin levels. The treatment also increased the number, diameter, volume and area of the pancreatic islets of diabetic rats [105] (2) The leaf gel (20, 30 and 50 mg/mL) and its phytosterols (lophenol, 24-methyl-lophenol, 24-ethyl-lophenol, cycloartanol, and 24-methylene-cycloartanol) showed anti-diabetic activity in type 2 diabetic mice. The phytosterols also reduced the HbA1c levels in mice in a 28 days study [104]
Zingiberaceae					
*Curcuma longa* L.	Haridra (S), Haldi (H), Turmeric (E)	Herb (C)/rhizome	Curcumin (22), demethoxycurcumin, bisdemethoxycurcumin, ar-turmerone [106]	(1) A small bit of raw rhizomes is chewed empty stomach (2) The powder of dried rhizomes (1 teaspoon) or paste of fresh rhizomes (1 teaspoon) is used in food preparations	(1) The volatile oil obtained from the rhizome inhibited α-glucosidase enzymes more effectively than the acarbose (a standard drug) in vitro. A major volatile constituent (ar-turmerone) also had potent α-glucosidase (IC_50_ = 0.28 μg) and α-amylase (IC_50_ = 24.5 μg) inhibition [107] (2) Curcumin, a phenolic compound isolated from the rhizome reduced blood glucose and the levels of glycosylated haemoglobin in diabetic rats through the regulation of the polyol pathway. It suppressed oxidative stress and inflammation, and also suppressed increased bone resorption through the inhibition of osteoclastogenesis and expression of the AP-1 transcription factors, c-fos and c-jun, in diabetic animals. It showed a beneficial role in the diabetes-induced endothelial dysfunction and induced a down-regulation of nuclear factor-kappa B. It has a protective role against advanced glycation as well as collagen cross-linking [108]
*Curcuma zedoaria* (Christm.) Roscoe	Sugandha moola (S), Kachoor (H), Zedoary/White turmeric (E)	Herb (C)/rhizome	Curcumin (22), furanodiene, furanodienone, zedorone, curzerenone, curzeone, germacrone, 13‐hydroxy germacrone, dihydrocurdione, curcumenone, zedoaronediol [109]	The powdered rhizome (1 teaspoon) is taken with water twice a day for once a month	The methanol extract of rhizomes (200 and 400 mg/kg) reduced the serum glucose level in glucose-loaded mice using a GTT assay [110]
*Hedychium spicatum* Sm.	Shati (S), Kapur Kachari (H), Spiked ginger lily (E), Seerh (L)	Herb (C)/rhizome	Essential oil (mainly contains 1,8‐cineole α- and β‐pinene, linalool, 10‐epi‐γ‐eudesmol and β‐slinene), hedychenone, spicatanol (**10**), 6-oxo-7,11,13-labdatrien-16,15-olide, picatanol methyl ether, hedychenone, 7-hydroxy hedychenone, yunnacoronarin A and D, 7-acetoxy hedychenone, hedychia lactone B [111]	The powdered rhizome (1 teaspoon) is taken with water twice a day for once a month	(1) Spicatanol isolated from rhizomes displayed rat intestinal α-glucosidase inhibitory activity in vitro by 89% at a dose of 100 µg/mL [112] (2) The oral dose of essential oil obtained from rhizomes (0.3 mL for 14 days) reduced blood glucose and urea level in ALX induced diabetic rats. It was also observed that the Islets of Langerhans regained their normal shape after 14 days of treatment [113]
*Zingiber officinale* Roscoe	Ardarka/Moolaja sunthi (S), Adarakh (H), Ginger (E), Aaddo (L)	Herb (C)/rhizome	Gingerols, shogaol [114]	Rhizome in the form of a tea, juice or food is taken daily for a long time	The rhizome juice (4 mL/kg/day for 6 weeks) increased insulin levels and decreased fasting glucose levels in STZ induced type 1 diabetic rats. The juice also decreased serum cholesterol, serum triglyceride and blood pressure in rats [115]

*H* Hindi, *E* English, *A* Ayurvedic, *L* local, *S* Sanskrit, *C* cultivated, *W* wild

During the field survey, it has been noted that *Artemisia* sp., *C. pareira*, *J. adhatoda*, *S. virginianum* and *V. negundo* are commonly found in roadside areas from Sahiya to Chakrata. On the other hand, *B. aristata*, *M. philippensis* and *S. glabra* are the common plants in light forest areas. *T. chebula*, *T. bellirica* and *P. emblica* are found in edges of fields whereas *B. ciliata* is available at the rocky areas.

**Fig. 2:**
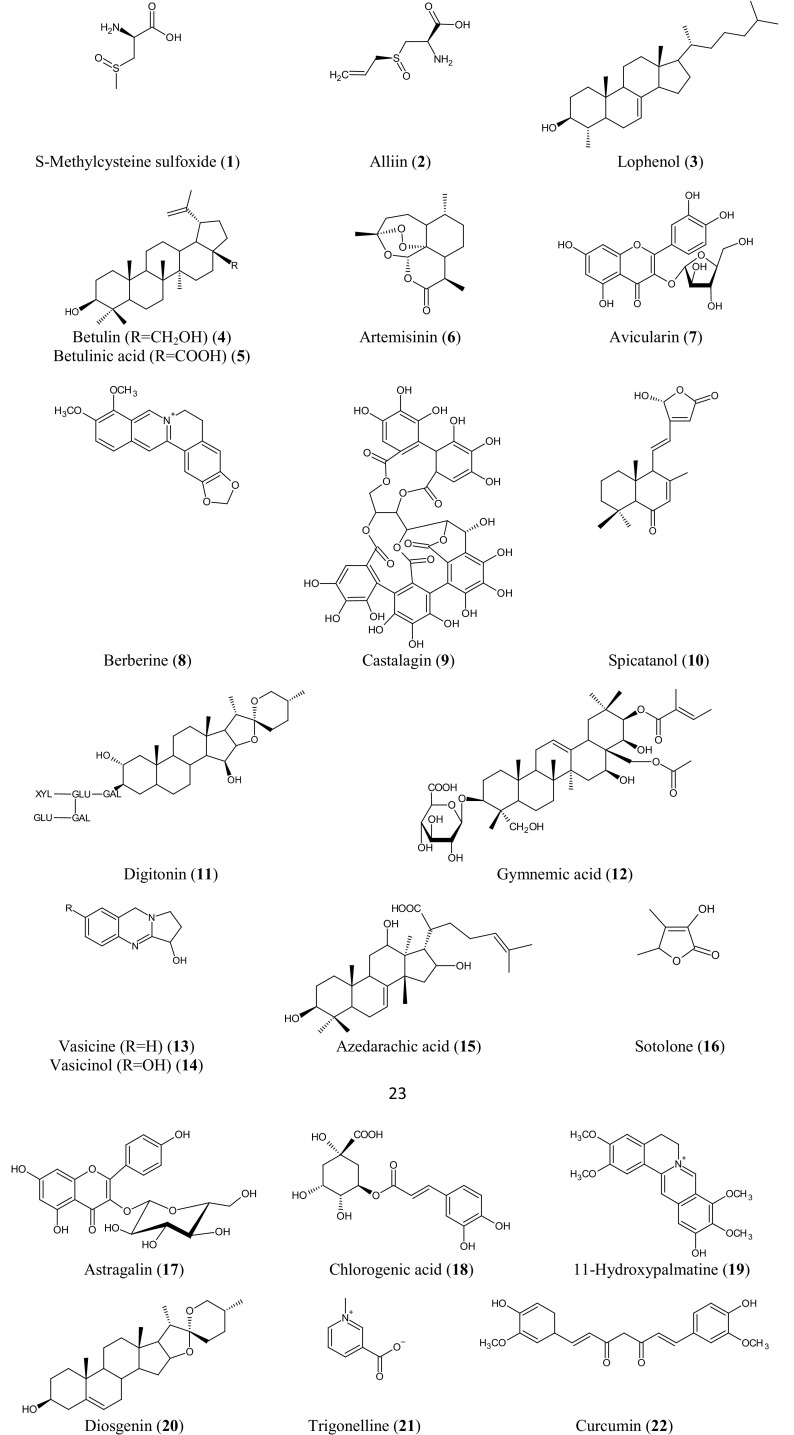
Selected antidiabetic compounds reported from surveyed plants

Various preclinical and clinical studies, as shown in Table 1, confirmed the role of surveyed plants in the diabetes mellitus. In addition to the crude extracts, various pure molecules have also been investigated as antidiabetic agents such as alliin, betulin, berberine, gymnemic acid and vasicine. Some mechanism-based studies also unravelled the involvement of different pathways in the antidiabetic activity of crude extracts or isolated molecules (Fig. 2).

## Existing Antidiabetic Formulations Prepared from Surveyed Plants

A number of medicinal plants found in the present study site are already used for the treatment of diabetes. Various formulations prepared from such plants are available in the market and frequently prescribed by Ayurvedic practitioners. Of these, few selected formulations based on the medicinal plants of the present survey are given in Table 2.

**Table 2:** Market formulations for diabetes treatment prepared from surveyed plants

Formulation	Ingredients
Amritamehari churna (classical medicine), Bio-gynema capsules, Glukostat capsules, Diabegon capsules, Debix, Diaxinol (patent drugs)	*G. sylvestre*
Chandraprabha vati (classical drug)	*B. aristata*, *C. deodara*, *C. longa*, *H. vulgare*, *P. emblica*, *T. bellirica*, *T. chebula*, *T. sinensis*, *Z. officinale*, *A. heterophyllum*
Dhanvantaram-ghritam (classical medicine)	*M. philippensis*
Diabeco (patent drug)	*O. tenuiflorum*
Diabecon (patent drug)	*B. aristata*, *C. longa*, *G. sylvestre*, *P. emblica*, *T. bellirica*, *T. sinensis*, *A. vera*, *A. racemosus*
Diacare capsules (patent drug)	*C. longa*, *P. emblica*, *O. tenuiflorum*, *T. chebula*
Diasulin (patent drug)	*C. longa*, *G. sylvestre*, *P. emblica*, *T. sinensis*, *T. foenum*-*graecum*
Fenfuro (patent drug)	*T. foenum*-*graecum*
Garlic capsules (patent drug)	*A. sativum*
Karnim plus (patent drug)	*Z. officinale*, *A. indica*, *O. tenuiflorum*
Kishora guggulu (classical medicine)	*P. emblica*, *T. bellirica*, *T. chebula*, *T. sinensis*
Limit capsules (patent drug)	*T. foenum*-*graecum*, *G. sylvestre*
Mandoor-Vatak	*C. deodara*
Neem capsules (patent drug)	*A. indica*
Nisakathakadi kashayam, Nishamalaki chunra (classical medicine)	*C. longa*
Sarivadyasava (classical medicine)	*F. religiosa*, *H. spicatum*
Triphla (classical medicine)	*P. emblica*, *T. bellirica*, *T. chebula*

## Market Demand for Selected Surveyed Plants

Presently, herbal drugs and cosmetics have a huge market worldwide mainly in Asian countries. In India, the National Medicinal Plants Board (Govt. of India) is the whole sole agency for monitoring and documenting the Indian medicinal plants. The agency is involved in the cultivation and promotion of medicinal plants to ensure the availability of quality raw material for the manufacturing of herbal drugs and cosmetics.

*A. heterophyllum*, commonly known as Atis or Ativisha, has about 100–200 metric tons annual trade in India. The plant is mainly cultivated in India, Nepal and Pakistan. Due to its diverse therapeutical applications in microbial infections, fever, vomiting, coughs, diarrhoea, and indigestion, it is used as one of the ingredients of many Ayurvedic formulations including Bala chaturbhadrika churna, Chandraprabha vati, Khadiradi gutika, Kutaj ghan vati, Lakasminarayana rasa, Mahavisagarbha taila, Panchkrita guggulu ghrita, Rasnairandadi kashayam, Rasnairandadi kvatha churna, Rodhrasava, Siva gutika and Sudarshana churna, etc. [116]. Kachnar (*B. variegata*), which is used in wounds, ulcer, thyroid problems, cervical lymphadenitis and rectal prolapse, is an important ingredient of Kachnar guggulu, Chitrakadi taila, Chandanasava, Ushirasava, Gandamala kandana rasa and Mutra sangrahaniya kwata. According to the National Medicinal Plants Board, Ministry of AYUSH, Government of India, its current market demand is also about 100–200 metric tons annually. Talispatra (*T. baccata*) and Devadaru (*C. deodara*) are also important Ayurvedic medicinal plants and their annual market demand is 100–200 and 1000–2000 metric tons, respectively.

Devadaru (*C. deodara*), an Ayurvedic remedy for inflammation, fever, pruritus, infested wounds, allergic rhinitis, constipation, drowsiness, hiccups, diabetes, cough, skin disease, rheumatoid arthritis and blood disorders, has been used in the preparation of khadirarista, dasamularista, devadarvarista, mrtasanjivanisura, karpuradyarka, pramehamihaira taila, chandanadi churna, narayana taila, pradarantaka lauha, vataraktanaka lauha, mahavisagarbha taila, anu oil, maharasnadi kashayam, devdarvadi kashaya, chandraprabha vati and pushkaramoolasav formulations. On the other hand, *T. baccata*, useful in cancer, blood disorders, skin disease, burning sensation, worms and papules, is an ingredient of Mahanarayana Taila and Bala Taila. *U. dioica*, helpful in kidney stones, allergies, anaemia, osteoarthritis, hay fever, burns, internal bleeding, nosebleeds, urination problems, gout, sciatica, neuralgia, haemorrhoids and hair problems, is a part of various formulations including vishatinduka taila and caffeine-free herbal tea. Its market demand is comparatively lesser (about < 10 metric tons annually) than other important Ayurvedic herbs.

In view of the market demand, a number of antidiabetic plants are cultivated in India including in the present study area. The main cultivated medicinal plants of the study area are *Z. officinale*, *C. longa*, *A. cepa*, *A. sativum*, *A. graveolens*, *C. zedoaria*, *H. vulgare* and *T. foenum*-*graecum*. The trade of *Z. officinale* and *C. longa* rhizomes in Indian market is about 2000–5000 and 1000–2000 metric tons, respectively. *Z. officinale* is used in the formulation of Trikatu churna, Ardraka khandavaleha, Sarassvatarista, Adraka ghrita, Shothaghna lepa, Saubhagya shunti and Guladrakm. India is known as one of the largest producers of ginger and turmeric in the world. Particularly in Chakrata, both of these plants are cultivated at large scale and considered as an important source of income. Similarly, other medicinal plants such as *A. vera*, *P. emblica* (> 10,000 metric tons), *T. chebula* (5000–10,000 metric tons), *M. piperita*, *A. racemosus*, *A. indica*, *J. adhatoda*, *T. bellirica* (2000–5000 metric tons), *O. tenuiflorum* (2000–3000 metric tons), *B. aristata*, *T. sinensis*, *R. cordifolia*, *B. ciliata*, *O. gratissimum* (1000–2000 metric tons), *S. virginianum*, *T. foenum*-*graecum*, *V. negundo*, *G. sylvestre* (500–1000 metric tons), *H. spicatum*, *H. vulgare*, *A. cepa*, *A. sativum*, *Z. armatum*, *C. roseus*, *C. zedoaria*, *F. religiosa* (200–500 metric tons), *B. prionitis*, *R. emodii* and *M. azedarach* (100–200 metric tons) are cultivated in the study region with high market demand.

## Mechanism of Actions of Surveyed Plants and Their Bioactives

Various earlier research studies reported the possible mechanisms of action shown by the antidiabetic plants as well as their bioactive constituents described in the present paper. *A. vera* and its phytosterols, i.e. lophenol, 24-methyl-lophenol, 24-ethyl-lophenol, cycloartanol and 24-methylene-cycloartanol shows glucose lowering effect through effectively decrease gluconeogenesis and lipogenesis processes. This activity predominantly appears to be mediated by AMPK and PPAR receptors and is accompanied mainly by inhibition of PEPCK and G6P genes expression or inhibition of ACC and FAS enzymatic activities hence reduce hepatic glucose output [104]. Moreover, the hypoglycemic effect of aloes and other bitter principles may be mediated through stimulating synthesis and/or release of insulin from the β-cells of Langerhans [117].

Berberine, an active principle of *B. aristata* was found to alter glucose metabolism through the stimulation of glycolysis via increasing glucokinase activity, increasing insulin secretion, and suppressing hepatic gluconeogenesis and adipogenesis [118–120]. Similarly, a bioactive molecule *S*-methycystein sulphoxide found in *A. cepa* regulates the enzyme Glucokinase/Hexokinase and stimulates glucose utilization and insulin secretions [121]. On the other hand, quercetin, another bioactive of *A. cepa* increases glucose-stimulated insulin secretion through an ERK1/2 pathway which improves liver and pancreas functions by enabling the recovery of cell proliferation through the inhibition of Cdkn1a expression [122].

*Curcuma longa* and its active molecule curcumin increase the islet viability and delay islet ROS production in animals [123]. Curcumin treatment also increases the number of small pancreatic islets and decreases lymphocyte infiltration in pancreatic islets [124]. The intervention of a curcumin-rich extract improves β-cell functions, indicated by an increased HOMA-β and reduced C-peptide in a study conducted by Chuengsamarn et al. [125]. The molecule itself plays antioxidant defence by induction of the expression of HO-1, glutathione subunit, and NAD(P)H:quinone oxidoreductase 1 and increased basal insulin secretion in human islet [126].

*Artemisia roxburghiana* as well as its bioactives, such as artemisinin, betulinic acid and betulin, act as protein tyrosine phosphatases inhibitor. Through this mechanism, they are known to enhance insulin receptor phosphorylation and stimulate glucose uptake into cells. On the other hand, its flavonoids inhibit cAMP phosphodiesterase which is a modulator of insulin secretion [37]. *G. sylvestre* has been reported to interact with glyceraldehyde-3-phosphate dehydrogenase, a key enzyme in the glycolysis pathway. In addition, its extract stimulates insulin secretion from human islets [127] and regenerates pancreatic β-cell in diabetic animals [128]. A leaves extract containing gymnemic acids as a bioactive suppresses the elevation of blood glucose level by inhibiting glucose uptake in the intestine [129]. Gulfraz et al. [15] reported that *J. adhatoda* extract increases the level of insulin in diabetic rats. It brings antihyperglycaemic action perhaps by potentiation of pancreatic secretion of insulin from β-cell of islets or due to enhanced transport of blood glucose to peripheral tissue.

**Fig. 3:**
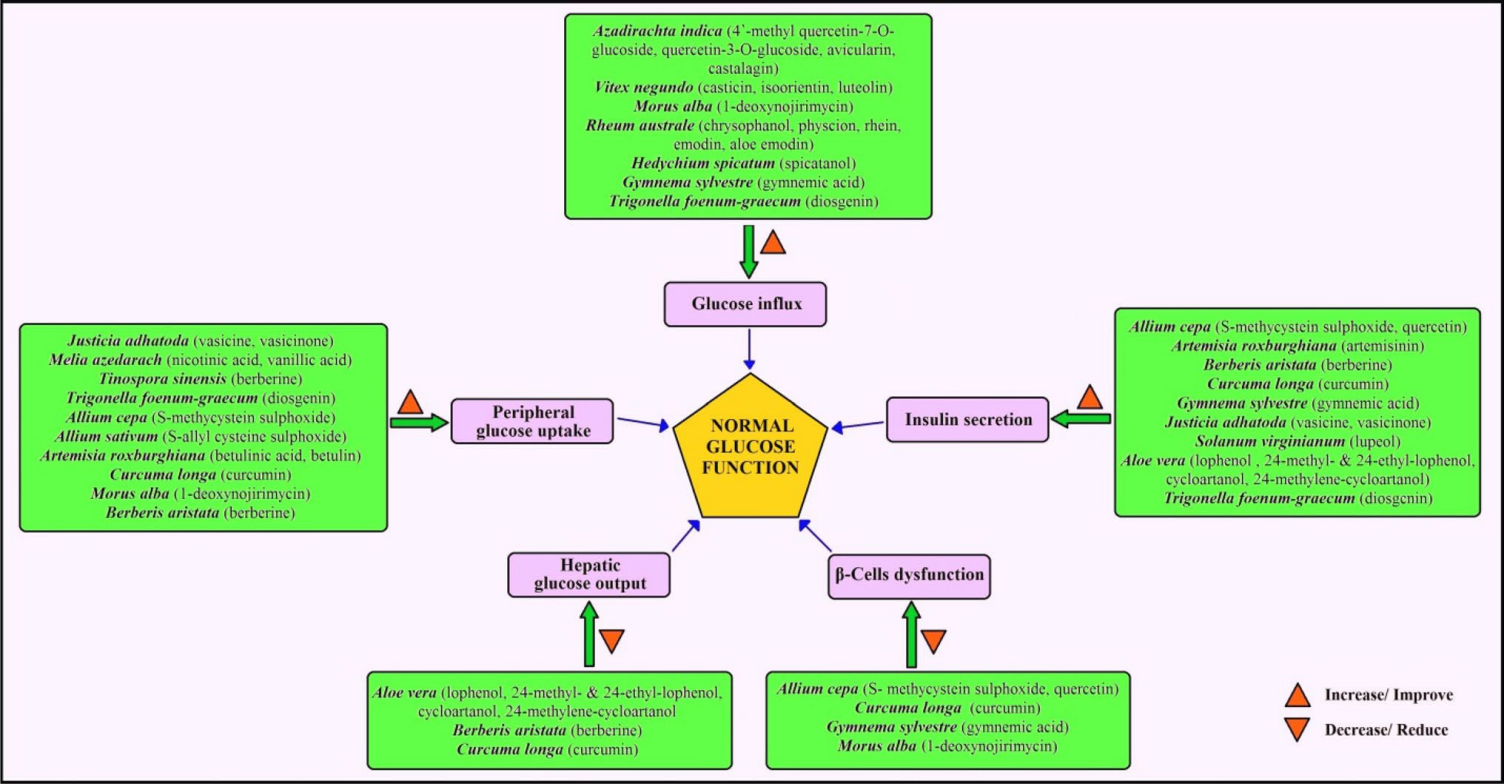
Schematic diagram showing antidiabetic mechanisms of selected surveyed plants and their bioactives

*Morus alba* has found to possess antihyperglycemic and antihyperlipidemic activities by activating the IRS-1/PI3K/Glut-4 signalling pathway in skeletal muscles and hence increases peripheral glucose uptake [130]. It interferes with the activity of α-amylase that exhibited an effective strategy to lower the levels of hyperglycemia through the control of starch breakdown [131]. In addition to inhibiting α-amylase, it reduces glucose level via scavenging of ROS and regenerating β-cell [132]. *M. azedarach* decreases blood glucose via inhibition of protein tyrosine phosphatase 1B (PTP1B) enzyme activity and stimulation of glucose uptake rats [133]. *T. sinensis* was also found to inhibit PTP1B activity and stimulate the glucose uptake [134]. *R. australe* and its active molecules rhapontigenin, desoxyrhaponticin and desoxyrhapontigenin inhibit the mammalian α-glucosidase [135], whereas *V. negundo* inhibits α-amylase to decrease blood glucose level [136]. *T. foenum*-*graecum* stimulates insulin secretion at all levels of the cellular organization since the cells are more sensitive to insulin and increase in the number of insulin receptor sites to burn cellular glucose at high fibre diet in rats [137]. A schematic diagram of the antidiabetic mechanisms of selected plants/bioactives is shown in Fig. 3.

## Status of the Medicinal Plants of Chakrata Region

Various plants in the Chakrata region are rare and only grown as wild, their cultivation is still a big challenge. The surveyed plants were checked in the IUCN Red List [138] for their current status and found that many of them are already included in the list of threatened species. According to the IUCN List, *B. variegata*, *C. deodara*, *T. baccata* and *U. dioica* are identified as the plants of least concern whereas *A. heterophyllum* and *S. hexandrum* are reported as an endangered plant. Although many rare plants, including *A. heterophyllum*, are cultivated in a big scale in India and exported to other countries [139], many other plants of this particular region are also in the stage of vulnerable and their protection is highly needed. Various reports published in past years also highlighted the importance of these plants including their medicinal uses, market demand and current status [140–143].

## Conclusion

The Chakrata region (Jaunsar–Bawar Hills) is one of the sites of the rich diversity of medicinal plants in Uttarakhand, India. Most of the areas of this region are covered by the forests with a very scattered population mainly the tribal community. The population is mainly depends on folk medicine due to limited hospitals in the region. These people prefer to consult the local traditional healers or elderly person in the family for the treatment, and only moved to the distantly located hospitals in case of any serious health trouble.

There are various nurseries, such as Deovan and Khoonigarh, in the region established by the Forest Department (Govt. of Uttarakhand) to cultivate valued medicinal and other economic plants including some fruits and timber yielding plants. In addition, the natives also cultivate several useful plants to generate the economy for their survival. There is good support of the local government to encourage them by providing quality seeds, fertilisers and tools in subsidised rates.

The present study site has numerous antidiabetic plants found as wild such as *B. aristata*, *J. adhatoda*, *C. pareira*, *S. glabra*, *A. roxburghiana*, *T. baccata*, etc. This region is also popular for cultivating many rare medicinal plants such as *A. heterophyllum*, *S. hexandrum* and *B. variegata*. Thus, with the help of cultivation in a big scale, the threatened plants in this region can be conserved for the future. The overharvesting of the medicinal plants can also be minimised by investigating their bioactive compounds and synthesising them in the laboratories to fulfil the market demand of such plants. This approach may also be worthy in case a particular medicinal plant becomes extinct in the future, its drug/formulation will be always available for the treatment of a particular disease.

## Abbreviations

STZ: Streptozotocin
ALX: Alloxan
GTT: Glucose tolerance test
